# Preoperative multimodal ultrasonic imaging in a case of Peutz-Jeghers syndrome complicated by atypical lobular endocervical glandular hyperplasia: a case report and literature review

**DOI:** 10.1186/s13053-024-00275-7

**Published:** 2024-02-28

**Authors:** Liwen Yang, Duan Duan, Ying Xiong, Tianjiao Liu, Lijun Zhao, Fan Lai, Dingxian Gu, Liuying Zhou

**Affiliations:** 1 Department of Ultrasonography, School of Medicine, Chengdu Women’s and Children’s Central Hospital, University of Electronic Science and Technology, No. 1617, Riyue Avenue, Chengdu, Sichuan 610091 China; 2 Department of Gynecology, School of Medicine, Chengdu Women’s and Children’s Central Hospital, University of Electronic Science and Technology, Chengdu, China; 3 Department of Obstetrics, School of Medicine, Chengdu Women’s and Children’s Central Hospital, University of Electronic Science and Technology, Chengdu, China

**Keywords:** Peutz-Jeghers syndrome, Multimodal ultrasonography, Atypical lobular endocervical glandular hyperplasia, Gastric-type endocervical adenocarcinoma, Contrast-enhanced ultrasonography, Three-dimensional ultrasonography

## Abstract

**Background:**

Peutz-Jeghers syndrome (PJS), an autosomal dominant multiple cancerous disorder, is clinically characterized by mucocutaneous macules and multiple gastrointestinal hamartomatous polyps. Gastric-type endocervical adenocarcinoma (G-EAC), a special subtype of cervical adenocarcinoma with non-specific symptoms and signs, is known to occur in approximately 11% of female patients with PJS.

**Case presentation:**

Here, we report a case of PJS in a 24-year-old female with multiple mucocutaneous black macules who complained of vaginal discharge and menorrhagia. Moreover, we first described the multimodal ultrasonographical manifestations of PJS-correlated G-EAC. The three-dimensional reconstructed view of G-EAC on 3D realisticVue exhibited a distinctive “cosmos pattern” resembling features on magnetic resonance imaging, and the contrast-enhanced ultrasound displayed a “quick-up and slow-down” pattern of the solid components inside the mixed cervical echoes. We reported the multimodal ultrasonographical characteristics of a case of PJS-related G-EAC, as well as reviewed PJS-related literature and medical imaging features and clinical characteristics of G-EAC to provide insight into the feasibility and potential of utilizing multimodal ultrasonography for the diagnosis of G-EAC.

**Conclusions:**

Multimodal ultrasound can visualize morphological features, solid components inside, and blood supplies of the G-EAC lesion and distinguish the G-EAC lesion from normal adjacent tissues. This facilitates preoperative diagnosis and staging of PJS-related G-EAC, thereby aiding subsequent health and reproductive management for patients with PJS.

## Synopsis

We reported multimodal ultrasonographical characteristics of a case of Peutz-Jeghers syndrome-related gastric-type endocervical adenocarcinoma (G-EAC), indicating the potential use of multimodal ultrasonography for G-EAC diagnosis.

## Background

Peutz-Jeghers syndrome (PJS) is a rare, autosomal dominant, multiple-organ cancerous syndrome clinically characterized by widespread mucocutaneous hyperpigmented macules and multiple hamartomatous polyps across the gastrointestinal tract. PJS has a low incidence, impacting only one in 50,000 to 200,000 individuals [1]. In childhood, symptoms are mainly caused by polyp-related complications, including bleeding, anemia, and obstructive symptoms of the digestive tract. Patients with PJS have a substantial risk of developing diverse cancers in adulthood, most of which have an early onset [2–4]. By the age of 70, patients with PJS have a cumulative cancer incidence rate of 85% [3]. Patients with PJS have a substantially increased risk of gastrointestinal cancer, with cumulative incidence rates of 39, 29, and 13% for colorectum, stomach, and small intestinal cancer, respectively [5]. Female patients with PJS may develop various gynecological tumors, including sex cord tumors with annular tubules (SCTAT), lobular endocervical glandular hyperplasia (LEGH), ovarian mucinous tumor, endometrioid adenocarcinoma, gastric-type endocervical adenocarcinoma (G-EAC), approximately 50% of which correlated with PJS syndrome [6].

However, given that PJS is a rare syndrome and multilocular cervical lesions of G-EAC appear similar to deep Nabothian cysts, missed diagnosis or misdiagnosis remains a possibility. Herein, we report a case of PJS in a 24-year-old female with multiple mucocutaneous black macules who complained of vaginal discharge and menorrhagia. Moreover, we first described the multimodal manifestations of PJS-correlated G-EAC. We retrieved and analyzed previous reports on the ultrasonic diagnosis of G-EAC; these reports mainly focused on the gray-scale ultrasound (US) and color Doppler US and described G-EAC sectional morphological features and blood supplies [7–10]. We found that by using the multimodal US, we were able to show the overall 3D morphological structure; measure cervical-lesion volume, blood supply, and vessel distribution; quantify VI, FI, and VFI values of the cervical-mass blood supply; and visualize the potential solid components inside the cystic components, which is critical for distinguishing the malignant and non-malignant nature of the cervical mass. A hysteroscopic biopsy revealed gastric mucous glandular lesions in the cervix. A diagnosis of PJS was suspected based on the patient’s medical history, medical imaging features, and mucocutaneous macules, and the patient was advised to undergo laparoscopic exploration and cervicectomy. Postoperative pathological examination confirmed the presence of atypical LEGH (aLEGH) in resected cervical tissues. Moreover, we reviewed available literature regarding PJS and medical imaging features and clinical characteristics of G-EAC to comprehensively clarify PJS and G-EAC and avoid a missed diagnosis or misdiagnosis by gynecologists, radiologists, and sonologists.

## Case presentation

**Fig. 1:**
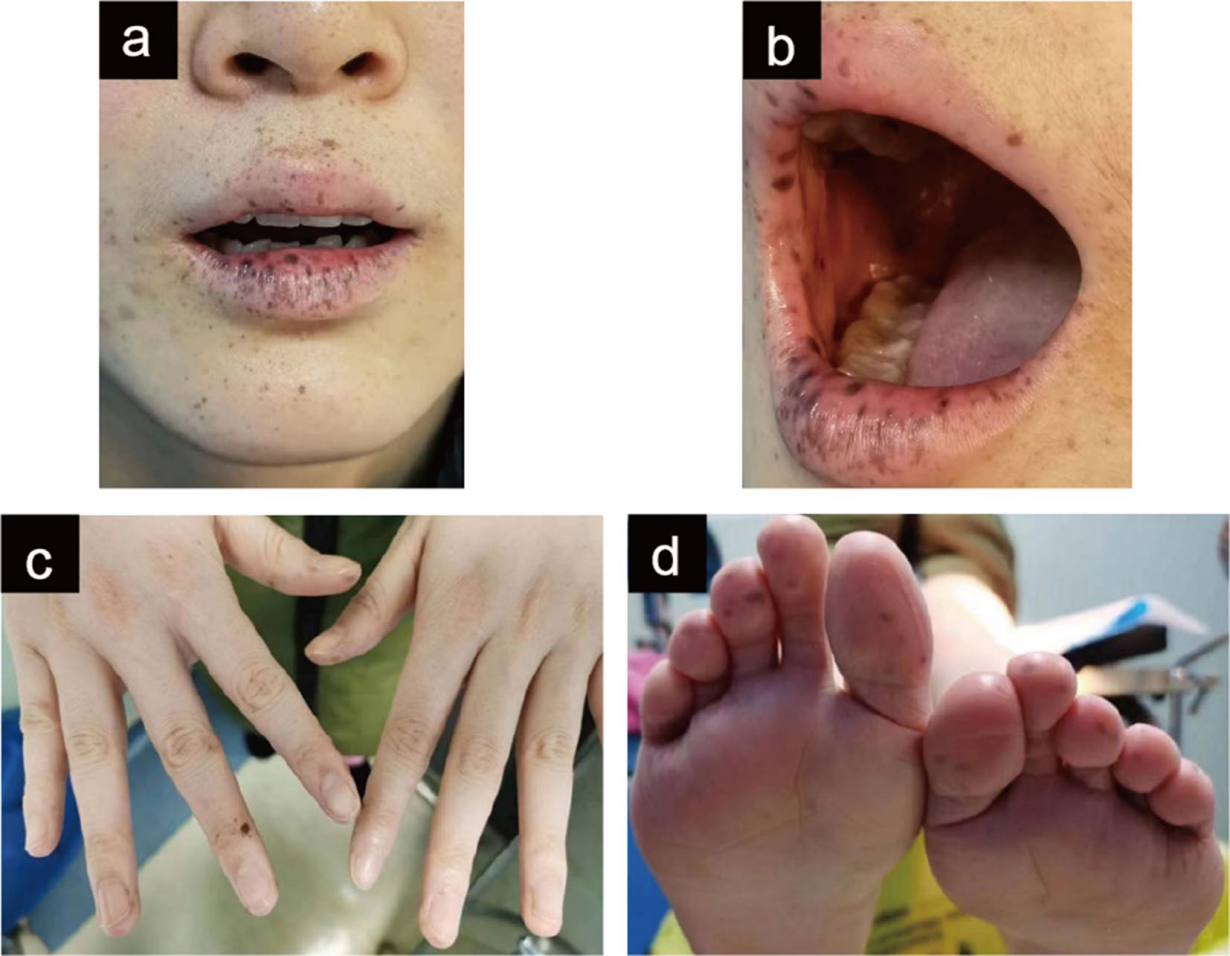
Multiple hyperpigmented macules on the lips (**a**), buccal mucosa (**b**), fingertips (**c**), and toes (**d**) of the patient

**Fig. 2:**
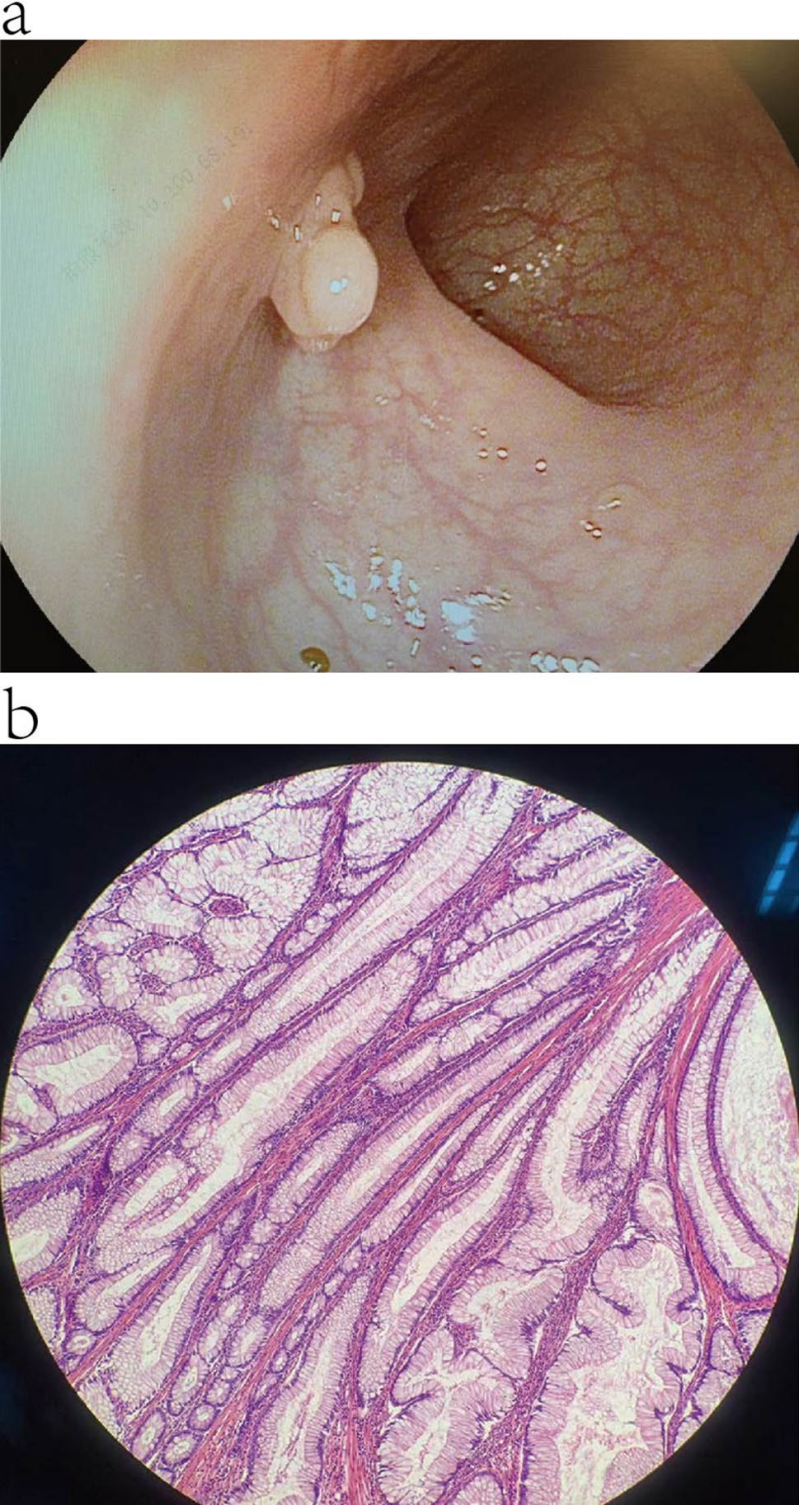
Polyp-like neoplasms in patient’s gastrointestinal track. (**a**) Gastrointestinal endoscopy reveals multiple polyp-like neoplasms. (**b**) histopathological examination of polyp resected from the mid-ileum

A 24-year-old (gravida 0 para 0) female without experience of sexual intercourse was referred to our gynecology department with complaints of menorrhagia and vaginal discharge. Physical examination revealed multiple hyperpigmented macules on her lips, buccal mucosa, fingertips, and toes (Fig. 1). She was diagnosed with mild anemia at 10 years of age; genetic screening for thalassemia reported no abnormalities. At 15, she underwent a laparotomy to treat an “intestinal obstruction.” Since the age of 21 years, she has experienced a prolonged menstrual period (50–60 days) and hypermenorrhea, accompanied by a clean and odorless vaginal discharge. The US examination showed an enlarged cervix with multicystic lesions, the largest of which measured 1.9 cm×3.4 cm×2.2 cm. The patient has since experienced occasional hematochezia and was referred to several medical institutions to treat abnormal uterine bleeding. At 23 years of age, she underwent gastrointestinal endoscopy, revealing chronic non-atrophic gastritis and multiple polyps in the stomach, duodenum, and rectum. The pathological diagnosis of the resected polyp specimen from the mid-ileum revealed chronic active inflammation involving the superficial mucosa (Fig. 2). During this period, the medical imaging examination revealed a growing cervical mass. Serum gynecological tumor markers, including alpha-fetoprotein (AFP), carcinoembryonic antigen (CEA), cancer antigen (CA) 199 (CA199), CA153, and CA125, were within normal levels. The human papillomavirus screening test results for cervical secretions were negative. The patient’s father and younger brothers had similar black mucocutaneous macules, and her grandfather died of gastrointestinal bleeding. Except for her father, who was also diagnosed with leukoderma, the patient and her family denied the presence of other familial diseases.

**Fig. 3:**
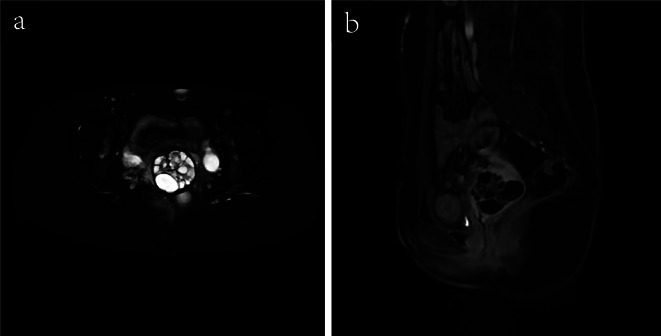
MRI examination of cervical lesions. (**a**) Cross-sectional plane on the T2WI fat suppression sequence exhibits an enlarged cervix with multilocular space-occupying lesions of various sizes abutted through irregular septae. (**b**) The sagittal plane of contrast-enhanced MRI reveals the noticeably enhanced cyst walls and septae. *Abbreviation* MRI, magnetic resonance imaging

**Fig. 4:**
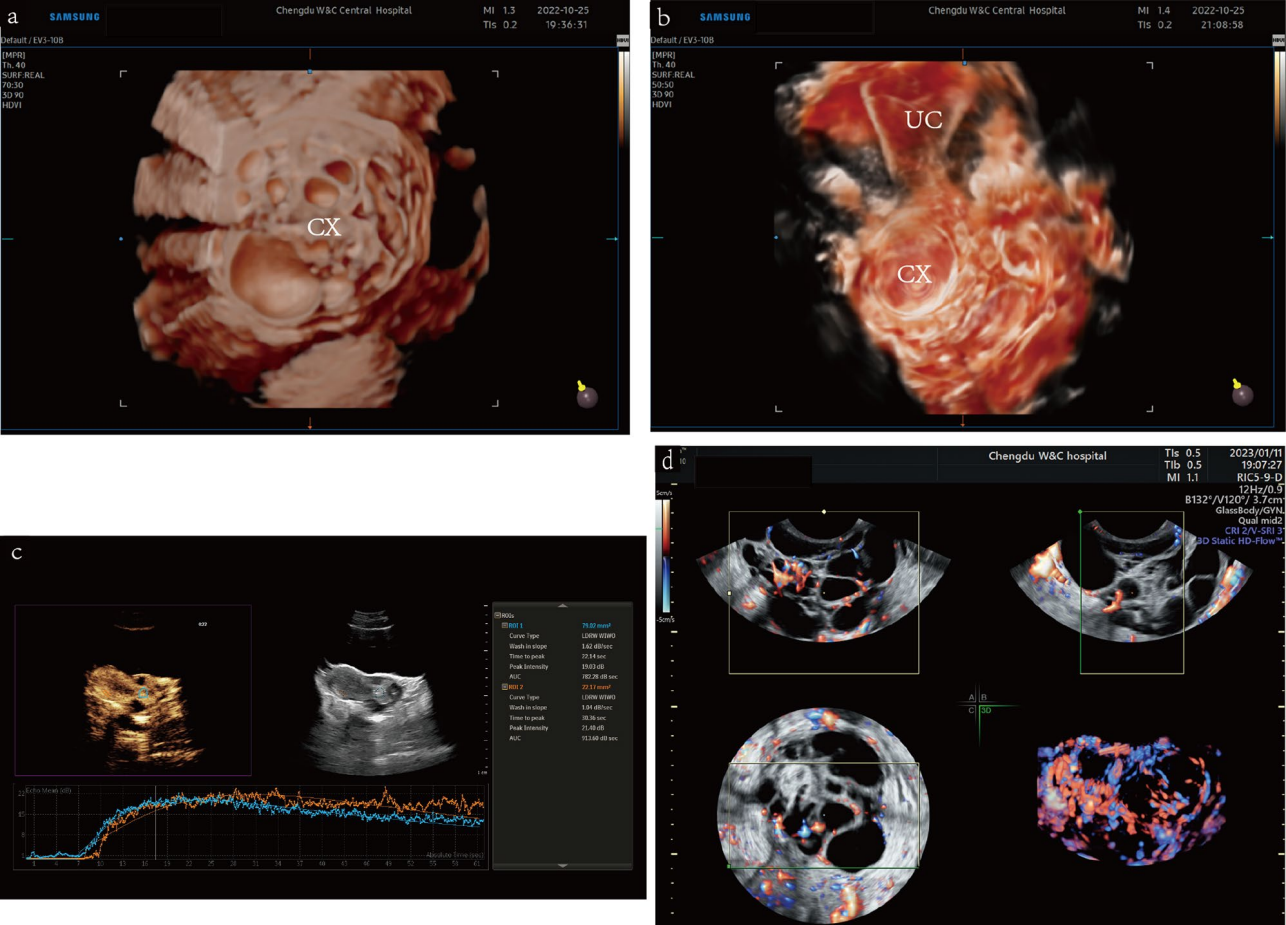
Three-dimensional and contrast-enhanced ultrasonography imaging. (**a**) Three-dimensional view on 3D realisticVue (Samsung Medical, Seoul, South Korea) shows multilocular signs resembling the “cosmos pattern” in MRI. (**b**) Three-dimensional reconstructed coronary section of uterus and cervix presented by the CrystalVue software (Samsung Medical, Seoul, South Korea). The enlarged barrel-shaped cervix with multilocular resonances and an uninvaded uterine cavity can be observed. (**c**) CEUS (PHILIPS EPIQ7, Philips Healthcare, Seattle, WA, the United States) examination on the blood perfusion of the cervical lesion. The ROI and curve in blue indicate the perfused region and TIC of the solid component in the cervix; the ROI and curve in orange represent the perfused region and TIC of the myometrium. The solid components of the cervical mixed echo began developing at the 7th second and peaked at the 22nd second, presenting a pattern of “quick-up and slow-down.” (**d**) Three-dimensional power Doppler (General Electric Company, Boston, Massachusetts, the United States) displays the increased blood supplies with irregular ramification, part of which multiplies and forms a clump-like pattern. *Abbreviation* CEUS, contrast-enhanced ultrasonography; CX, cervix; EN, endometrium; MRI, magnetic resonance imaging; ROI, region of interest; TIC: time-intensity curve

**Fig. 5:**
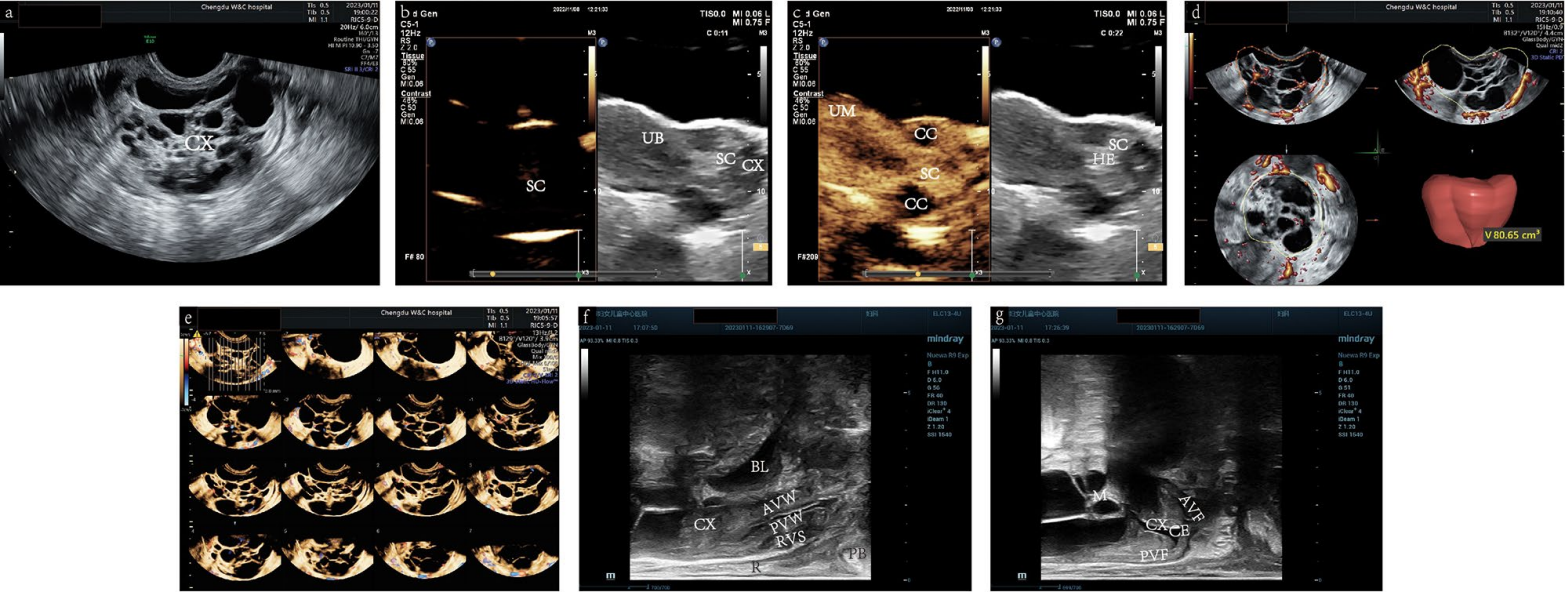
Multimodal ultrasonography of cervical lesions. (**a**) Transrectal gray-scale US (GE voluson E10 RIC5-9-D, General Electric Company, Boston, Massachusetts, the United States) showing an enlarged cervix with multiple cystic lesions of various sizes and a few solid components. The solid components are hyperechoic. The cystic lesions are irregular, honeycomb-shaped, and of various sizes and were encompassed by relatively larger cysts. (**b**) Transabdominal CEUS ((PHILIPS EPIQ7, Philips Healthcare, Seattle, Washington, the United States) showing perfusion of the solid components of the cervical lesions before the myometrium. (**c**) Transabdominal CEUS image showing an unevenly enhanced uterine myometrium, unenhanced cystic echoes, and equally enhanced solid components when peaked. (**d**) Measurement of the mass volume using VOCAL software. (**e**) Three-dimensional TUI (General Electric Company, Boston, Massachusetts, the United States) of blood flow in the cervical lesion indicates that its blood supply is located in the intercyst septate and solid components. (**f**) Biplane transrectal US sagittal view using Biplane Endocavity convex-linear array transducer (ELC13-4U, Mindray, Shenzhen, Guangdong, China) revealing the normal structure of the vagina without penetration of the cancerous lesion. (**g**) Biplane transrectal US sagittal view showing intact and smooth rims of the anterior and posterior vaginal fornices without thickening or penetration of the lesion. *Abbreviation* AVF, anterior vaginal fornix; AVW, anterior vaginal wall; AVW, anterior vaginal wall; BL, bladder; CC, cystic component; CE, cervical effusion; CEUS, contrast-enhanced ultrasonography; CX, cervix; HE, hyperecho; PB, perineal body; PVF, posterior vaginal fornix; PVW, posterior vaginal wall; PVW, posterior vaginal wall; R, rectum; RVS, rectovaginal septum; SC, solid component; TUI, tomographic ultrasound imaging; UM, uterine myometrium; US, ultrasound

After admission to the inpatient department, the patient underwent magnetic resonance imaging (MRI) (Fig. 3) and multimodal US (Figs. 4 and 5). Transrectal US revealed an enlarged, barrel-shaped cervix (6.4 cm×5.7 cm×6.0 cm) containing multilocular lesions of various sizes comprising a few solid components. The cervical lesions occupied almost the entire cervix, with the upper rim reaching the internal cervical os and the lower rim 0.7 cm above the external cervical os. No cervical myometrial echo was found. Some solid components were found in the septae between neighboring cysts, which altogether measure 4.0 cm×3.0 cm×3.3 cm; the cystic-solid echoes were encompassed by relatively larger cystic echoes. The cystic echoes, with diameters of 0.1–1.0 cm, were honeycomb-shaped with a slightly higher solid echo. The solid echo and septae showed strip-like rich blood flow signals with an arterial spectrum resistive index of 0.57. The cervical canal had a 0.4 cm separation, making the line of the cervical mucosa invisible. No abnormal echoes were observed in the anterior or posterior vaginal fornix or in the endometrium and myometrium of the uterus. No space-occupying lesions were observed in the uterine parametrium. An anechoic cyst, measuring 3.4 cm×2.3 cm×2.8 cm, was found in the left ovary. The ovarian cyst had a thin wall and was surrounded by a few short, strip-like blood flow signals. No lesions were observed in the right adnexa. The three-dimensional reconstructed view using 3D realisticVue (W10 EV3-10B, Samsung Medical, Seoul, South Korea) displayed multilocular lesions resembling the “cosmos pattern” in MRI examination, nearly occupying the whole cervix [11]. Three-dimensional power Doppler US revealed an increased blood supply with irregular ramifications, parts of which multiplied and formed clump-like patterns. Three-dimensional tomographic US imaging of the blood flow of the cervical lesion indicated that its blood supply was located in the intercyst septae and solid components. The blood flow histograms revealed the following results: vascularization index, 4.567; flow index, 35.374; vascularization-flow index (VFI), 1.615. Using the VOCAL software (Voluson ^TM^ E10 BT19, General Electric Company, Boston, Massachusetts, the United States), the volume of the mass measured was 80.65 cm.^3^ Contrast-enhanced ultrasonography (CEUS) (PHILIPS EPIQ7, Philips Healthcare, Seattle, WA, the United States) using intravenous SonoVue (Bracco, Milan, Italia) revealed that solid components of the cervical mixed echo begun to develop at 7 s and peaked at 22.14 s, which gradually faded and presented a slightly increased signal in the final stage, exhibiting a “quick-up and slow-down” pattern of the time-intensity curve. CEUS also displayed a local density random walk wash-in/wash-out curve type with a wash-in slope of 1.62 dB/s, peak intensity of 19.03 dB, time from peak to 1/2 of 39.67 s, and mean transit time of 26.63 s. The cervical serous layer displayed continuous echoes of the signal, which did not increase simultaneously with the solid components of the cervical masses. The MRI revealed an enlarged cervix with multilocular space-occupying lesions of various sizes involving the entire muscular layer.

**Fig. 6:**
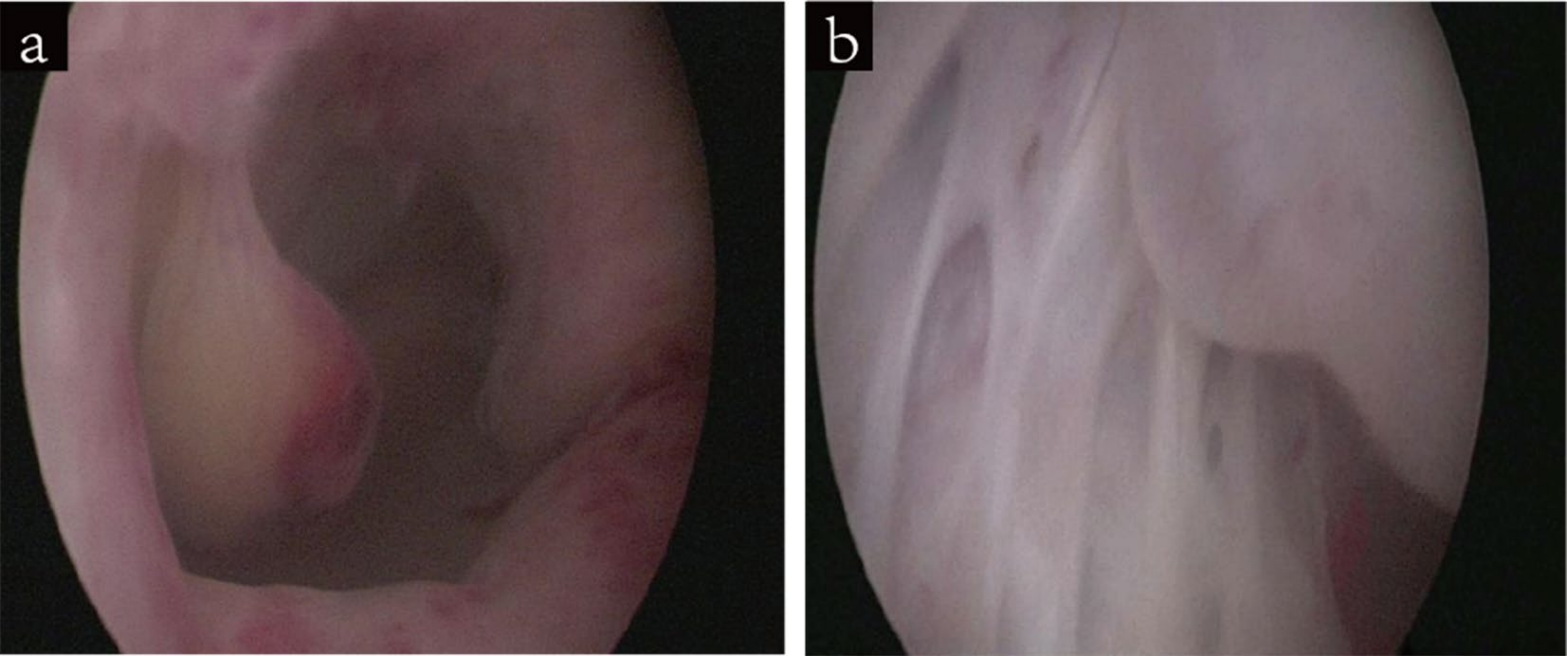
Hysteroscopic views of the uterine cavity and cervix. (**a**) Presence of multiple polyp-like neoplasms in the uterine cavity. (**b**) Presence of multilocular lesions in the cervix

**Fig. 7:**
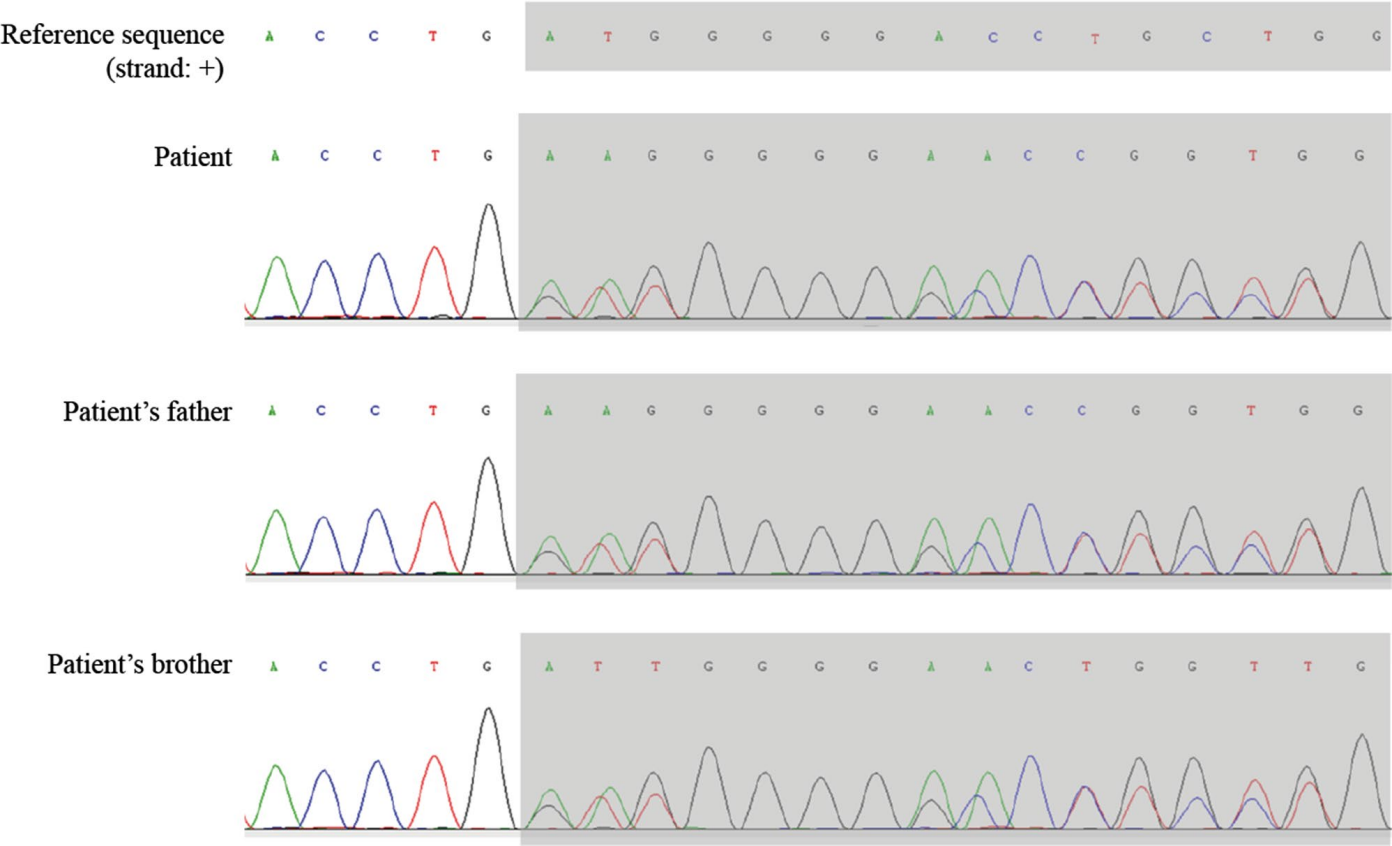
Sequencing results for *STK11* in the patient and her family members. Whole-exome sequencing (patient) and Sanger sequencing (patient’s father and brother) confirmed the pathogenic heterogeneous mutation of the *STK11* gene mapped to Chr19:1207063–1,207,063, NM_000455.5:exon1:.150dup (p.Met51Aspfs*112)

Subsequently, a hysteroscopic biopsy revealed multiple polyp-like neoplasms in the uterine cavity and multilocular lesions in the cervix (Fig. 6). Pathological examination of the biopsy specimen revealed LEGH and aLEGH. Whole-exome and Sanger sequencing confirmed the *STK11* gene mutation in the patient and her family (Fig. 7; Table 1).

**Table 1:** *STK11* sequencing results in the patient and her family members

Sample	Gene	Site(hg38)	Reference	Alteration	Mutant type	Alleles
Patient	*STK11*	Chr19:1207062	-	G	Heterozygous	-/G
Patient’s father	*STK11*	Chr19:1207062	-	G	Heterozygous	-/G
Patient’s brother	*STK11*	Chr19:1207062	-	G	Heterozygous	-/G

**Fig. 8:**
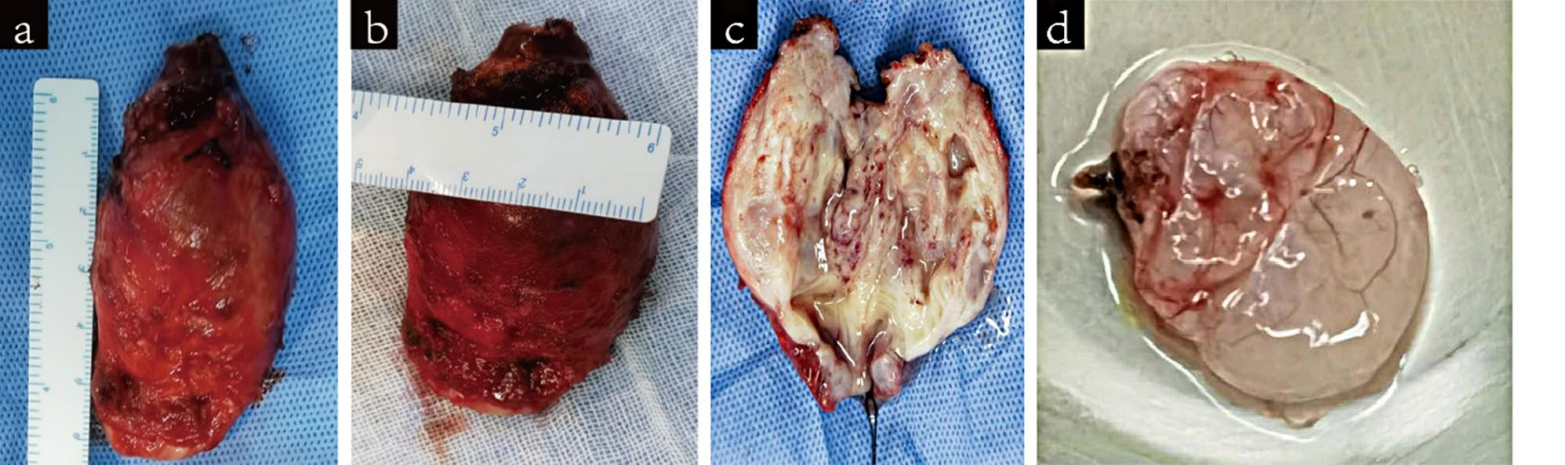
Macroscopic view of the resected tissues. (**a**-**b**) The enlarged cervix, measuring 6 cm × 5 cm × 4 cm, exhibits a barrel-like shape; (**c**) the inside view of the cervix presents multiple cystic cavities with smooth walls, which contain a large amount of water-like clean mucous fluid; (**d**) Macroscopic view of the resected left ovarian cyst measured 5 cm × 4 cm × 3 cm. The cyst exhibits a thin wall and watery fluid inside

**Fig. 9:**
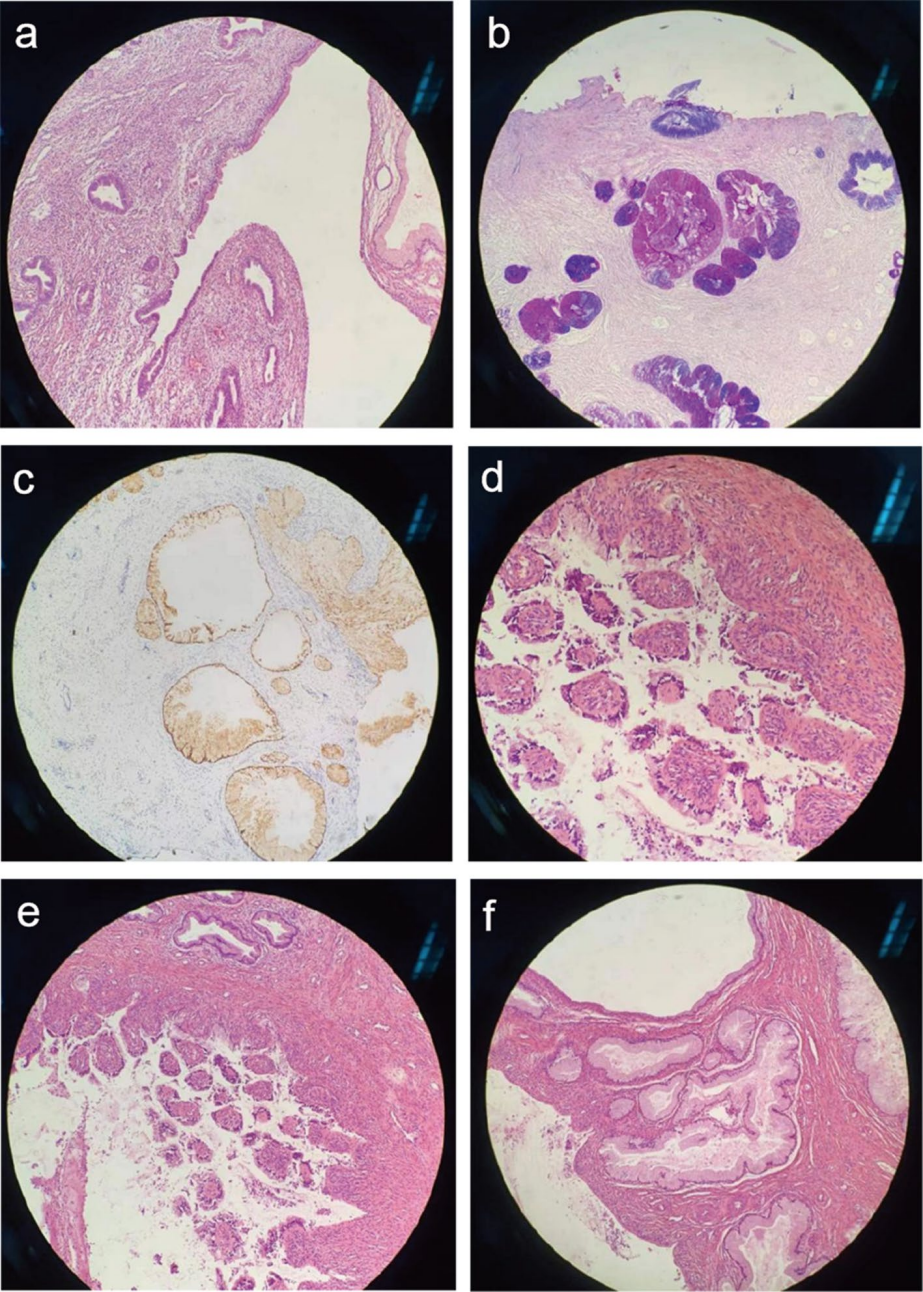
Postoperative pathohistological examination of resected cervical tissue. (**a**) The glandular lesion invading the cervix and lower part of the uterus; (**b**) AB/PAS staining shows the neutral mucus inside of the gastric-type cells; (**c**) Positive staining of MUC6 by IHC, indicating the glandular epithelium was gastric-type; (**d** & **e**) the presence of LEGH in the cervix; (**f**) The presence of SGM and LEGH in the cervix. *Abbreviation* AB/PAS, Alcian-blue/Periodic acid Schiff reagent; IHC, immunohistochemistry; LEGH, lobular endocervical glandular hyperplasia; SGM, simple gastric metaplasia

Based on the evidence mentioned above, the patient was diagnosed with PJS and recommended undergoing laparoscopic exploration and cervicectomy according to clinical guidelines [2, 12]. The lesion-penetrated parts of the lower uterus, cervix, and ovarian cyst were resected and subjected to pathological examinations, revealing that the cervix and lower part of the uterus were both invaded by glandular lesions (Figs. 8 and 9). Immunohistochemistry revealed Polyclonal Antibody to Mucin 6 (MUC6)-positivity. Simple gastric metaplasia (SGM) and LEGH in the cervix were also documented, suggesting the presence of gastric-type glandular epithelium. Alcian-blue/Periodic acid Schiff reagent (AB/PAS) staining revealed the presence of neutral mucus in the gastric-type cells.

## Discussion

Given that patients with PJS have a considerably increased risk of developing cancer in multiple organs, the early diagnosis of PJS and concurrent cancer and timely surgical intervention are critical for improving patient prognosis. Gynecological tumors are commonly observed among female patients with PJS. G-EAC is the most common type of non-HPV-associated cervical adenocarcinoma known to occur in approximately 11–17% of female patients with PJS [13, 14], accounting for nearly 10–15% of all cervical adenocarcinomas [15], accompanied by non-specific clinical manifestations. Except for physical examinations, genetic screening, patients’ medical history of gastrointestinal polyps, and family history of PJS, the application of medical imaging might help a lot in diagnosing PJS-induced G-EAC. Herein, we reported a case of PJS in a 24-year-old woman complicated with G-EAC by mainly focusing on the medical imaging features, especially multimodal ultrasonographical manifestations of the G-EAC lesions, and reviewed the available literature regarding the epidemiology, clinical characteristics, diagnostic criteria, and methods, focusing on medical imaging, and management of PJS and G-EAC.

## Clinical features, diagnosis, and treatment of PJS and G-EAC

PJS affects approximately one in 100,000 individuals and is characterized by black macules in the labial regions, oral cavity, and extremities, as well as multiple gastrointestinal polyps [16, 17]. PJS is attributed to a mutation in the gene encoding serine-threonine kinase 11 tumor suppressor and is autosomal dominant [18, 19]. Accordingly, PJS is also called familial mucocutaneous hyperpigmented gastrointestinal polyposis. Females with PJS may develop gynecological tumors, which include various pathologic types, such as SCTAT, LEGH, G-EAC, ovarian mucinous tumor, and endometrioid adenocarcinoma [6].

The diagnosis of PJS can be established if any one of the following criteria is met: (1) at least two histologically confirmed PJ polyps; (2) at least one histologically confirmed PJ polyp and family history of PJS in close relatives; (3) pigmented moles on skin and mucosa and family history of PJS in close relatives; (4) at least one histologically confirmed PJS polyp and pigmented moles on skin and mucosa [12]. A diagnosis of G-EAC is mainly based on pathological examinations combined with immunohistochemical staining. Notably, PJS and G-EAC frequently cooccur in a single individual; therefore, if PJS or G-EAC is diagnosed or suspected, the possibility of the other should be carefully examined. A pelvic MRI should be conducted in a timely manner. Moreover, a deep cervical biopsy under US guidance, segmented curettage, and cervical conization should also be performed when necessary [20].

For the management of PJS, the surveillance of gastroenterological polyps and the occurrence of cancer is of marked relevance, as no radical therapy is available for curing PJS [12]. Owing to age-dependent features of PJS, the early diagnosis of sizable gastrointestinal polyps and timely surgical interventions can avoid intussusception and anemia in young patients. Regular screening and surgical removal of cancerous lesions at early stages can substantially improve the prognosis of adult patients.

Since no standard therapy has been established for addressing G-EAC, personalized treatment is highly recommended for individual patients. During the early stages of G-EAC, the removal of cervical lesions and metastases, if any, by surgical intervention is critical. Furthermore, chemoradiotherapy should be undertaken postoperatively. For advanced-stage G-EAC, chemoradiotherapy remains the predominant treatment method [20, 21]. Reportedly, the expression of human epidermal growth factor receptor-2 (HER-2) is commonly observed in patients of G-EAC with ovarian metastasis or in advanced stages; hence, targeted therapy using HER-2 monoclonal antibodies, such as trastuzumab, has been deemed a feasible therapeutic option for G-EAC [22].

### Medical Imaging Features and its applications in the diagnosis of G-EAC

During early-stage G-EAC, the macroscopic appearance of the cervix is relatively normal, as the lesion is hidden in the middle and upper part of the cervical canal, making it markedly difficult to be sampled during routine screening. Moreover, owing to the mildly heterotypic cytological feature of G-EAC, the positive rate of G-AEC in cervical exfoliative cytology and cervical biopsy examinations is only 32.7% [23]. Therefore, conventional cervical cancer screening methods fail to meet the clinical needs for diagnosing this disease. These factors highlight the importance of medical imaging in diagnosing G-EAC and distinguishing benign and malignant cervical lesions using supplementary methods [7, 24, 25].

We briefly summarized the major features of G-EAC examined by different medical imaging modalities in Table 2. Given the low soft tissue contrast resolution, computed tomography (CT) is infrequently applied to diagnose G-EAC. Under CT imaging, G-EAC presents an enlarged cervix comprising masses of low intensity, most of which are multiple complex cystic masses with smooth edges, and a few are solid masses with blurred edges [7, 8, 26–28]. Contrast-enhanced CT can provide more details and even reveal cystic lesions with enhanced contrast, which cannot be detected under ordinary CT examination.

**Table 2:** Features, advantages and disadvantage of different medical imaging methods on G-EAC

Modalities	Medical imaging features of G-EAC	Advantages	Disadvantages
CT	Enlarged cervix comprising masses of low intensity, most of which are multiple complex cystic masses with smooth edges, and a few are solid masses with blurred edges	Enhanced CT could show the cystic lesions with enhanced contrast Able to manifest the regional invasion and distant metastasis of the cervical lesion	Low soft-tissue contrast; High radiation dosage; Relatively low resolution of the solid component inside of the cervical mass;
MRI	Cosmos sign; Barrel-like multicystic mass with hyper-intense in T2-weighted images, comprising solid components that grow from the cervical glands into the stroma, replacing the original fibromuscular cervical stroma	High soft-tissue contrast; Able to exhibit the solid components inside of the cervical masses; Capable of assessing the regional invasion of the cervical lesion	Relatively pricy and time-consuming; Not suitable for patients with metal prosthetic, Implantable cardioverter-defibrillators (ICDs), or pacemaker, or end-stage renal disease:
Gray scale US and color doppler US	Enlarged cervix with multilocular cystic lesions, multilocular cystic lesions with solid components inside, or purely solid lesions; Increased blood supply in the cystic septae between neighboring cysts or the solid component of the cervical mass under color doppler US	Non-invasive,inexpensive,and widely available; Able to show the blood supply of the cervical lesion	Only able to show the sectional images and blood supply of the lesion rather than the overall morphology and blood supplies; Unable to measure the volume,blood supply and vessel distribution of the lesion; Incapable of distinguishing the solid invasive lesion from the normal cervical tissue,lower resolution in showing the invasion into the adjacent structure or distant metastasis of the cervical mass as compared with MRI and contrast enhanced-CT thus has limited diagnostic value
Multimodal US	3D realisticVue exhibited a distinctive “cosmos pattern”; blood flow histogram of the cervical mass shows increased VI, FI, and VFI values; CEUS shows that the solid components inside of the cystic-solid mixed components of the cervical mass increased prior to the cervical myometrium, presenting a equal-high enhanced pattern.	Able to show the overall morphological structure, Measure the volume, blood supply and vessel distribution of the cervical lesion; Capable of quantifying the VI, FI, and VFI values of the blood supply of the cervical mass; Capable of visualizing the potential solid components inside of the cystic components and quantifying the CEUS parameter of the solid components; Able to assess the presence of invasion into the adjacent structure and distant metastasis; Better capability of assessing the vaginal invasion than MRI	Time-consuming; More expensive than oridinary US; Worse than CT in finding distant metastasis

According to certain specialists, MRI may better reflect the histological architecture of the cervical lesion, detect its solid components, and present more detailed features than CT [8, 24, 26, 29]. Based on the pathomorphological and distribution patterns of lesions, the typical MRI patterns of G-EAC were classified into four types including “cosmos”(small cystic components encompassed by bigger cysts), “diffuse growth”(multiple cysts of small size and diffuse distribution), “focal mass-like bulging”(unilaterally distributed numerous microcysts forming a focal bulge that grows outward of the cervical wall), and “solid and cystic”(bilaterally distributed multiple cysts of various sizes and mixed components). The first two types are deemed benign lesions; the third type is precancerous, whereas the last type indicates malignancy [30]. Typically, in most cases, G-EAC exhibits barrel-like multicystic massed with hyper-intense in T2-weighted images, comprising solid components that grow from the cervical glands into the stroma, replacing the original fibromuscular cervical stroma [24]. In certain cases, G-EAC lesions in the T2 image may be purely solid or cystic [23, 28, 31]. In contrast, the postcontrast T1-weighted images exhibit fat saturation, revealing the mildly heterogeneous enhancement of the solid components inside the cervical lesion [24]. Importantly, the solid lesion might be correlated with elevated risks of invasion and metastasis, which, accordingly, is a critical feature for distinguishing the malignancy of the multilobular cystic masses because non-malignant masses merely contain solid components inside [28, 32]. Moreover, consistent growth of the cervical lesion during adulthood might indicate precancerosis and even cancerization [25]. Like the case reported herein, the growing size may also indicate the malignant nature or tendency of the lesion.

US is considered the first-line screening method for cervical cancer owing to its low price, convenience, and high reproducibility. Gray-scale US images of G-EAC show an enlarged cervix with multiple internal masses in the upper part of the cervix with multilocular cystic lesions, multilocular cystic lesions mixed with solid components, or purely solid lesions [10]. Occasionally, the lower part of the uterine body may be invaded. The multilobular cyst is commonly observed in gray-scale US, which shows multiple cystic or solid-cystic echoes of various sizes with smooth or blurred edges surrounded by larger cystic echoes. The color Doppler US can reveal the moderate or abundant blood flow signals of G-EAC [7–10, 33].

Interestingly, the three-dimensional reconstructed views of the G-EAC on 3D realisticVue markedly resemble the “cosmos pattern” in MRI [11]. Three-dimensional energy Doppler US can visualize the blood flow in the lesion and its spatial position in a three-dimensional structure. Moreover, it can measure the volume of the mass and display the vascularization index (VI), flow index (FI), and VFI [33], which are valuable for distinguishing between benign and malignant cervical masses, as malignant masses tend to have increased volume, uneven distribution of blood supply (some blood flows may increase and show a clump-like pattern), and increased VI, FI, and VFI values [34, 35]. CEUS can exhibit the underlying solid component inside of the cystic component, which is critical for distinguishing the benign or malignant nature of the cervical lesion, whereas the Gray-scale US cannot discriminate the malignant nature of the solid component within the cystic component. Under CEUS, the solid components of the mixed echo developed prior to the uterine myometrium, showing equal-high enhancement, whereas the normal cervical stroma does not develop before the myometrium, showing low-equal enhancement, which is one of the evident features for distinguishing the benign or malignant nature of lesions. Moreover, the CEUS provides valuable quantitative parameters and facilitates preoperative lesion assessment [36, 37]. In the current case, the quantitative parameters of CEUS, such as arrival time (AT), time to peak (TTP), rise time, and mean transit time (MTT), were shorter than those of the myometrium, and peak intensity (PI) was slightly lower than the myometrium, which was equally enhanced. Given that CEUS of the current case displayed a “quick-up and slow-down” pattern of solid components inside the mixed cervical echoes in the TIC, which noticeably differed from the “quick-up and quick-down” pattern of cervical carcinoma [36], we concluded that the cervical lesions in this case may be precancerous. These phenomena could be explained by the increased neovascularization within the lesion, gradual decrease or even absence of smooth muscle cells and elastic fibers, and the loss of function of vascular endothelial cells. Accordingly, the contrast agent fills and regresses faster than normal tissues, leading to shortened AT, TTP, rise time, MTT, and elevated PI [28, 38, 39]. In addition, the CEUS can present different aspects of local invasion, including parametrial extension and invasion of the vagina, uterine corpus, and other adjacent organs. CEUS has good concordance with MRI in evaluating the invasion of cervical cancer [40]. In this case, echoes of the parauterine and uterine body did not enhance synchronously with the solid components of the cervical mass, indicating that they were not invaded by the cervical lesion. Besides, the echoes of the serous layer were continuous, suggesting that the rectum and bladder were not invaded. MRI and traditional US have been reported to have low sensitivity in diagnosing local invasion of the cervical lesion into the vagina (merely 44.4% by MRI and 55.6% by traditional US [41]. Accordingly, another study reported that MRI and the traditional US were not entirely reliable in assessing vaginal invasion of the cervical lesion [42]. Therefore, we performed bi-plane transrectal US—an emerging technique that applies a high-frequency probe with a frequency range of 3.2–12.8 MHz and has a higher resolution compared to ordinary vaginal US probes. Thus, the entire structure of the vagina, including the anterior and posterior fornix and part of the cervix, could be depicted, and whether the vagina has been invaded could be clearly demonstrated.

However, US-obtained imaging features of G-EAC share many similarities with those of other cervical multicystic lesions, such as deep Nabothian cysts, tunnel cluster, cervicitis, and cervical endometrial hyperplasia, all of which exhibit an enlarged cervix with multicystic lesions, occasionally with increased blood flow signals in the presence of inflammation. Therefore, diagnosing G-EAC using solely gray-scale and color Doppler US remains a challenge. Nevertheless, it is possible to differentiate G-EAC from other cervical multicystic diseases mentioned above based on imaging features of the multimodal US and clinical manifestations with relatively high accuracy. Benign diseases such as Nabothian cysts, tunnel plexus, and cervical endometrial hyperplasia exhibit very few solid components in most cases, reduced or no blood flow signals in the cystic septum and wall, and a clear rim; their VI, FI, and VFI values are also low, typically small in size, with low risk of invading the lower part of the uterine body and they barely invade the parametrium and vagina. Therefore, a preliminary diagnosis of Nabothian cysts can be reached if the patient has the aforementioned medical imaging features and a history of dyspareunia or a feeling of fullness in the vagina. Tunnel clusters could be suspected if the patient has corresponding imaging characteristics and a history of multigravida, although the final diagnosis should be based on the results of histological examinations. Given the abundant inflammation-induced blood flow, uterine cervicitis may exhibit malignant signs in some cases with no “cosmos pattern” on MRI. Moreover, clinical manifestations may include pelvic pain and pressure, accompanied by a yellowish jelly-like vaginal discharge with an unpleasant odor. Following drug treatment, the blood flow signals may drop to normal levels during follow-up. Accordingly, cervicitis could be suspected based on these medical imaging features and clinical manifestations. In addition, a high T2 signal intensity and iso-intensity in T1 found in the cervix of patients of reproductive age or a history of oral progesterone usage may suggest cervical endometrial hyperplasia [30, 43]. However, special types of cervical hyperplasia, such as LEGH, are precancerous lesions of G-EAC. The preoperative differential diagnosis of LEGH is difficult and requires further diagnosis by biopsy or histopathological analysis. If the mass grows during follow-up, surgical intervention should be considered.

In the current case of PJS-correlated G-EAC, owing to these qualitative and quantitative features of multimodal US, G-EAC was suspected before surgery, and clinical staging of the lesions was performed to provide valuable clues for operative decision-making. By preoperatively suspecting G-EAC using multimodal US findings in this case of PJS-related G-EAC, we aimed to highlight the usefulness of multimodal US, which combines two- and three-dimensional US and CEUS, in the diagnosis of PJS-related G-EAC and create awareness to prevent missed diagnosis or misdiagnosis of G-EAC by sonologists. To the best of our knowledge, this is the first report describing the multimodal ultrasonic manifestations and the CEUS features of PJS-associated G-EAC, and additional cases and clinical evidence are required to verify the specificity of our findings.

## Conclusions

Compared to the traditional gray-scale and color Doppler US, multimodal ultrasound can better visualize morphological features, solid components inside, and blood supplies of the G-EAC lesion and can distinguish the G-EAC lesion from normal adjacent tissues, facilitating preoperative diagnosis and staging of PJS-related G-EAC. The distinctive ultrasonographical features provided by the multimodal US can also serve as an early warning signal for PJS, contributing to improved subsequent health and reproductive management for both the patients and their families.

## Data Availability

All data generated or analysed during this study are included in this published article.

## Abbreviations

AB/PAS: Alcian-blue/Periodic acid Schiff reagent
AFP: alpha-fetoprotein
aLEGH: atypical LEGH
AT: arrival time
CA: Cancer antigen
CEA: carcinoembryonic antigen
CEUS: contrast-enhanced US
CT: Computed tomography
FI: flow index
G-EAC: Gastric-type endocervical adenocarcinoma
HER-2: human epidermal growth factor receptor-2
LEGH: lobular endocervical glandular hyperplasia
MRI: Magnetic resonance imaging
MTT: mean transit time
MUC6: Polyclonal Antibody to Mucin 6
PI: peak intensity
PJS: Peutz-Jeghers syndrome
SCTAT: sex cord tumor with annular tubules
SGM: simple gastric metaplasia
TTP: time to peak
US: Ultrasound
VFI: vascularization-flow index
VI: vascularization index
